# Online sexual abuse of adolescents by a perpetrator met online: a cross-sectional study

**DOI:** 10.1186/s13034-019-0292-1

**Published:** 2019-08-24

**Authors:** Linda S. Jonsson, Cecilia Fredlund, Gisela Priebe, Marie Wadsby, Carl Göran Svedin

**Affiliations:** 1 Barnafrid, Child and Adolescent Psychiatry, Department of Clinical and Experimental Medicine, Faculty of Medicine, Linköping University, 581 83 Linköping, Sweden; 2 Child and Adolescent Psychiatry, Department of Clinical and Experimental Medicine, Faculty of Medicine, Linköping University, 581 85 Linköping, Sweden; 3 Department of Psychology, Lund University, 221 00 Lund, Sweden

**Keywords:** Adolescent, Sexual abuse, Online, Health

## Abstract

**Background:**

The current study aimed at exploring adolescents’ experiences of online sexual contacts leading to online sexual abuse by a perpetrator whom the victim had first met online. Associations with socio demographic background, experience of abuse, relation to parents, health and risk behaviors were studied.

**Methods:**

The participants were a representative national sample of 5175 students in the third year of the Swedish high school Swedish (M age = 17.97). Analyses included bivariate statistics and stepwise multiple logistic regression models.

**Results:**

In total 330 (5.8%) adolescents had gotten to know someone during the preceding 12 months for the purpose of engaging in some kind of sexual activity online. Thirty-two (9.7%) of those, the index group, had felt that they had been persuaded, pressed or coerced on at least one occasion. Sexual interaction under pressure was seen as constituting sexual abuse. These adolescent victims of online sexual abuse, the index group, did not differ with respect to socio-demographic background from the adolescents without this experience, the reference group. The index group had significantly more prior experiences of different kind of abuse, indicating that they belong to a polyvictimized group. More frequent risk behavior, poorer psychological health, poorer relationships with parents and lower self-esteem also characterized the index group. Online sexual abuse, without experiences of offline abuse, was associated with a poorer psychological health, at least at the same level as offline sexual abuse only.

**Conclusions:**

The study made clear the importance of viewing online sexual abuse as a serious form of sexual abuse. Professionals meeting these children need to focus not only on their psychological health such as symptoms of trauma and depression but also need to screen them for online behavior, online abuse and other forms of previous abuse.

## Introduction

### Voluntary online sexual exposure

Most children in western countries use the internet daily [1]. Among 17 year olds in Sweden the figure is 98% [2]. The internet is mostly used for doing schoolwork, playing online games and watching film clips, but many young people also use it to stay in contact with people and to meet new people for friendship, love and/or sex [2, 3]. One behavior that has been well studied recently is that of young people sending or receiving nude images of themselves, so called sexting. The prevalence of sexting varies between 2.5 and 21% depending on definition of sexting and methodology used. Sexting is more common among girls than boys [4, 5]. In a Swedish study of 18-year-old students, 20.9% had engaged in some form of voluntary sexual exposure online by posting pictures of themselves partially undressed, flashing, masturbating, or having sex on webcam [6]. Similar results were reported by the same group from a study 5 years later where 21% of 18-year old students reported having posted or sent nude images [7]. The motivations for sexting have been found to sometimes be for reasons other than sexual; many individuals who engage in texting say they do it for fun, to receive confirmation, to be seen by other, or because they think it is expected from them by their partner when in a relationship. Sexting can also be done because a person has been threatened to send a nude image [8] in such cases an important boundary has been crossed into involuntary abusive situation.

### Online sexual abuse

Even if most sexual contacts online are voluntary and do not involve anything that might be seen as sexual abuse, there is always a possibility that children can be sexually abused online. One well studied area involving possible sexual abuse concerns unwanted sexual approaches, especially those made by an adult who contacts children for sexual purposes. In a Swedish study of 14–15 year old children, 30% (48% of the girls and 18% of the boys) reported that unknown adults had made contact with them via the internet and made suggestions of a sexual nature during the preceding year [9]. Sexual approaches were experienced more often by girls than boys and were also more common among older adolescents and those defining themselves as gay, bisexual or as being unsure about sexual orientation [7]. Wolak et al. [10] found that the group most vulnerable to sexual approaches and grooming tend to consist of high-risk youths with a prior history of sexual abuse. Individuals who use chatrooms, communicate with people met online, engage in sexual behavior online and who share personal information online also place themselves at risk [11–13]. Baumgartner et al. [14] found that adolescents taking most risks online also were more likely to face negative consequences such as abusive situations than those who did not engage in risky online behavior. These adolescents were more likely to be sensation seekers who have a low level of satisfaction with their lives and/or who have family difficulties.

Livingstone and Smith [15] found that fewer than one in five adolescents were affected by negative sexual experiences online. Hamilton-Giachritsis et al. [16] found in their study (including interviews and a questionnaire) of children victims of online sexual abuse, that the abuse involved control, permanence, black mail, re-victimization and self-blame. Among the participating children who were screened for post traumatic stress, four out of five had a score consistent with a diagnosis of posttraumatic stress. The study showed the seriousness of online sexual abuse and that the victims need professional support. Except for the study by Hamilton-Giachritsis et al. [16] the subject of online sexual abuse and the effects that follow have only been sparsely studied.

## Aim

The current study aimed to study experience that Swedish adolescents have had of sexual abuse by a person met online.

This study focused on the association of online sexual abuse with:

- Socio-demographic background
- Experiences of emotional-, physical- and sexual abuse
- Psychological health
- Relationships with parents
- Risk behaviors, including internet behavior.

## Methods

### Participants

The study population consisted of a representative sample of Swedish high school seniors in their third and last year at Swedish high school when most were 18 years old. In Sweden, about 91% of all 18-year-old adolescents are enrolled in high school [17]. The Swedish agency, Statistics Sweden, selected schools that might participate based on information from the Swedish National School Register. Stratification was made on the basis of school size and educational programs (20 programs ranging from those with a vocational profile to those designed to prepare students for entrance into a university) as indicated by data in the National School Register for second year high school student, in the fall term, 2013. One or two study programs were selected from each school.

A total of 13,903 adolescents from 261 of 1215 Swedish high schools were selected and of the 261 schools 238 met the criteria for selection in 2014. An additional sample from Stockholm County was selected using the same selection criteria. The response rate for Stockholm county was lower (48.7%) than for the rest of the country (65.3%). Differences were also seen regarding the size of schools. In Stockholm, fewer of the respondents came from schools with 10–190 pupils (13.9%) compared to the rest of the country (22.1%) and more often came from middle-size schools with 191–360 pupils (51.2%) compared to the rest of the country (41.6%), resulting in a small effect size (Cramer’s V = .10). Few differences were found between the sample from Stockholm and the rest of the country, so answers from Stockholm were used in this study.

Finally, 171 schools with 9773 adolescents agreed to participate in the study and 5873 students in these completed the questionnaire. Thirty-four questionnaires were excluded due to unserious answers or a high amount of missing data, leaving 5839 satisfactory questionnaires. This gave a response rate of 59.7%. The mean age of the participants was 17.97 (*SD* = .63). An additional 124 questionnaires were excluded since the index question, “Have you gotten to know anyone on the internet during the last 12 months that you had sex with online?” was not answered. The final sample consisted of 5715 adolescents. Participants who answered that they had felt persuaded, pressed or coerced when having sex online (sexually abused online) during the last year, constituted the index group and all other adolescents constituted the reference group.

### Procedure

The national agency Statistics Sweden distributed and collected the questionnaires. Information about the study was sent to the principals of the selected schools by mail in August 2014. Questionnaires were answered in digital format by entered answers into computers in 165 schools, where computers were not available, students filled in paper copies of the questionnaire (six schools). A reminder was sent to the schools that had not delivered data by the end of the first month. Information about the study was given to the principals and to the teachers in charge when the questionnaires were to be filled. Students gave their informed consent for participation by answering the questionnaire. All participating students received written information about where to turn for help and support if needed at any time after the day on which they had submitted the completed questionnaire.

### Measures

The questionnaire used in the present study was a modified version of a questionnaire used in two previous studies carried out in 2004 and 2009 (Svedin and Priebe [18, 19]). It comprised 116 main questions. Questions concerned socio-demographic background, experiences of abuse, and risk behaviors. In addition, three standardized instruments measuring relationships with parents and psychosocial health were used.

#### Socio-demographic background

Demographic questions were drawn up for the purpose of the study (listed in Table 2a). The adolescents self-reported the demographic information.

#### Abusive experiences

*Sexual abuse* was measured using the question: “Have you been exposed to any of the following against your will”, followed by six examples (someone flashed in front of you, touched your genitals, you masturbated someone, vaginal, oral, vaginal or anal penetration). The answers were analyzed in two categories, any sexual abuse (all questions) and penetrative abuse (oral, anal or genital penetration), see Table 2b.

*Emotional abuse* was measured using the question: “Have you prior to the age of 18 been subjected to any of the following by an adult”, with these three examples: been insulted, threatened to be hit, or been isolated from friends, see Table 2b. Participants who answered “yes” to one or more of the questions were considered victims of emotional abuse.

*Physical abuse* was measured using the same wording used for emotional abuse, but with eight examples of physical abuse (Table 2b). Participants who answered “yes” to one or more of the questions were considered victims of physical abuse.

#### Relationships with parents

The Parental Bonding Instrument [20, 21] is an instrument that measures an individual’s perception of parental styles during childhood. The instrument consists of 25 items, where 12 relate to the subscale “care” and 13 relate to the subscale “overprotection”. The response options are presented on a 4-point scale, from “very like” to “very unlike”. The total score for “care” ranges from 0 to 36 and from 0 to 39 for “overprotection”. Items assess perception of maternal and paternal behaviors separately. PBI has been evaluated as an attachment instrument with strong psychometric properties in a review by Ravitz et al. [22]. Cronbach’s alpha for mother care in the present sample was .87, and for father care .89. Mother and father overprotection were .84, and .78, respectively.

*Self*-*esteem* was measured by the Rosenberg self-esteem scale [23]. The instrument measures self-esteem using 10 items with four possible answers, ranging from “strongly agree” to “strongly disagree”. The total score varies between 0 and 30, with high scores corresponding to high self-esteem. In the current sample, Cronbach’s alpha for the total scale was .90.

*Trauma symptoms* were measured using the Trauma Symptom Checklist for Children [TSCC: 24, 25]. The questionnaire includes 54 questions that can be divided into six categories: anxiety, depression, post-traumatic stress, sexual concerns, dissociation and anger. Response options are “never”, “sometimes”, “often” and “almost all of the time”. Cronbach’s alpha in the present sample was .95 for the full instrument and .79–.88 for the six subscales.

#### Risk behaviors

Health-risk behaviors were measured using questions related to sexual or non-sexual risk-taking. Non-sexual risk-taking was measured with questions about use of alcohol and drugs, see Table 5.

*Sexual risk*-*taking behavior*s were measured using questions about age of onset for sexual debut and having had more than six sexual partners, see Table 5.

*Internet behavior* was measured with questions about time spent on the internet and seven questions mainly about sexual behavior on the internet during the last year, see Table 5.

*Pornography consumption* was measured by two questions, see Table 5.

### Data analyses/statistics

Bivariate statistical analyses were performed using Pearson’s Chi square statistics on categorical variables. Kolmogorov–Smirnoff test was performed to examine whether the PBI, Rosenberg, and TSCC scales (totals and subscales) could be assumed to be normally distributed. As these tests indicated that they were not normally distributed, bivariate analyses on these variables were performed using Mann–Whitney’s U test.

Furthermore, as there were too many variables to be included in a multiple logistic regression model, the number of variables to be included in a “final model” was reduced by performing stepwise multiple logistic regression analyses for each main table separately (each table identifies different group of factors that could be associated with sexual abuse on the internet, Table 4 excluded), Table 6.

All analyses were performed using SPSS, version 22.0 (IBM Inc., Armonk, NY). A *p* value < .05 (two-sided) was considered statistically significant.

### Ethics

The study was approved by the Regional Ethical Review Board of Linköping (Dnr, 131–31).

## Results

### Online sexual abuse

Of the total of 5715 students who answered the question about the experience of having sex online, 330 (5.8%) answered that they had had sex online on at least at one occasion during the preceding 12 months with  a person met online (Table 1). It was more common for boys than girls (8.3% vs. 3.7%, *p* < .001) to have had that experience, along with those who did not identify themselves as male or female (9.4%). Of the 330 students who had had sex online, 32 (9.7%), the index group, felt persuaded, pressed or coerced. It was more common for girls than for boys to have had the experience of sexual abuse online (12.8% vs. 7.2, *p *= .018).

**Table 1:** Online sexual abuse

	All n = 5715		Boy n = 2519		Girl n = 3143		Doesn’t fit n = 53		*p*-value
	n	%	n	%	n	%	n	%	*p*
Have you got to know anyone on the internet during the last 12 months that you had sex with online?									
No	5385	94.2	2311	91.7	3026	96.3	48	90.6	< .001^a,b^
Yes	330	5.8	208	8.3	117	3.7	5	9.4	
Yes, once	191	3.3	110	4.4	77	2.4	4	7.5	
Yes, several times	139	2.4	98	3.9	40	1.3	1	1.9	
Did you felt persuaded, pressed or coerced at any time?^c^									
No	298	90.3	193	92.8	102	87.2	3	60.0	.018^a^, ns^b^
Yes	32	9.7	15	7.2	15	12.8	2	40.0	

^a^Chi square test all groups

^b^Chi square test between boys and girls

^c^Of those who answered Yes on the first question

There was a difference in age between those in the reference group who had met a person online for a voluntary sexual experience (n = 298) and those in the index group. Those in the index group had more often met with older persons than for those in the reference group (78.1 vs. 53.4%, *p* = .007) who more often met someone of the same age.

### Sociodemographic background

The students in the index group generally had a slightly less favorable background as concerned these factors: parents more often unemployed and/or had a lower level of education, students did not live with their parents less often, less often took university-oriented study programs, more often had an immigrant background, and were more likely to have a poorer financial situation, than the students in the reference group. However, these differences were not statistically significant (Table 2a).

**Table 2:** Online sexual abuse—socio-demographic background (a) and experience of other forms of abuse (b)

	Not sexually abused on the internet N = 5258–5685		Sexually abused on the internet N = 30–32		p-value^a^
	N	%	N	%	*p*
a. Socio-demographic background					
Fathers working	4987	88.0	25	78.1	ns
Mothers working	4950	87.4	26	81.3	ns
Fathers with university education	2285	40.2	10	31.3	ns
Mothers with university education	2963	52.1	15	46.9	ns
Living situation					
With both parents or alternating	4058	71.4	19	59.4	ns
With one parent with or without a new partner	1208	21.3	12	37.3	ns
Alone with sibling or partner	377	6.6	1	3.1	ns
In foster care or institution	37	.7	0	.0	ns
Study program					
Theoretical	4047	71.2	20	62.5	ns
Immigrant background (self or at least one parent with immigrant background)	1574	27.7	13	40.6	ns
Family financial situation					
Good	4516	79.5	21	65.6	ns
Poor	981	17.3	8	25.0	ns
Don’t know	185	3.3	3	9.4	ns
b. Other forms of abuse					
Sexual abuse					
*Any sexual abuse*	1085	20.6	17	56.7	< .001
Only penetrative abuse	335	6.4	10	33.3	< .001
Emotional abuse					
*Any emotional abuse*	3276	57.8	26	81.3	.007
Insult	3100	54.7	24	75.0	.021
Threats of hitting	1113	19.6	18	56.3	< .001
Isolation from friends	938	16.5	15	46.9	< .001
Physical abuse					
*Any physical abuse*	1756	31.0	21	65.6	< .001
Pushed, shaken	1343	23.7	17	53.1	< .001
Hit with hands	814	14.4	16	50.0	< .001
Throw something	763	13.5	10	33.3	.005
Kick, bite, hit with fist	315	5.6	8	25.0	< .001
Strangle	208	3.7	7	21.9	< .001
Hit with objects	196	3.5	3	9.4	ns
Burn, scald	99	1.7	3	9.4	.019
Other physical assault	466	8.2	11	34.4	< .001

^a^ p-value based on Chi square or Fisher’s exact test

### Experience of other forms of abuse

As seen in Table 2b, students in the index group had been significantly more often exposed to different forms of abuse during their childhood than those in the reference group. For example, students in the index group were five times as likely to have experienced penetrative sexual abuse outside the internet than those in the reference group (33.3% vs. 6.4%, *p* < .001), and two times as likely to have had some kind of prior experience of physical abuse (65.6% vs. 31.0%, *p *< .001).

### Parental bonding, self-esteem and trauma symptoms

Table 3 shows that the students in the index group reported significantly poorer relationships with both their mothers and fathers than those in the reference group as indicated by experienced less parental care and more parental overprotection.

**Table 3:** Online sexual abuse—parental bonding (PBI), self-esteem (Rosenberg) and trauma symptoms (TSCC)

	Not sexually abused on the internet N = 5499–5659		Sexually abused on the internet N = 31–32		p-value^a^
	M	SD	M	SD	*p*
PBI					
Mother care	30.02	6.29	26.19	7.71	.002
Father care	27.88	7.43	21.10	7.58	< .001
Mother overprotection	11.69	6.82	16.32	7.72	.001
Father overprotection	10.60	6.63	16.26	7.09	< .001
Rosenberg	21.07	6.66	15.25	7.72	< .001
TSCC					
Anxiety	4.68	3.98	8.38	5.87	< .001
Depression	5.14	4.52	10.97	6.96	< .001
Anger	4.12	4.07	7.97	5.88	< .001
Posttraumatic stress	6.19	5.06	11.78	7.18	< .001
Dissociation	5.98	4.87	10.84	6.83	< .001
Sexual concern	2.23	2.48	4.72	3.98	< .001
Critical items	1.71	2.51	5.41	5.04	< .001
*Total score*	29.47	20.68	56.03	32.89	< .001

^a^ p-value based on Mann–Whitney U-test

Self-esteem measured by Rosenberg self-esteem scale was significantly lower in the index group than in the reference group (*M* = 15.25, *SD* = 7.72 vs. M = 21.07, SD = 6.66, *p* < .001), Table 3.

The students in the index group also reported having significantly poorer health on all subscales of the TSCC than those in the reference group (all *p* < .001), Table 3. Table 4 shows a more detailed description of the TSCC results. The students that had been sexually abused both online and offline scored higher than those abused only online, but the difference only reached significance on the subscale depression (*M *= 13.29, *SD* = 6.65 vs. 8.33, *SD.* = 7.43, *p *= .008). The index group scored generally higher on all scales than students abused outside the internet, but there were no statistically significant differences.

**Table 4:** Detailed description of trauma symptoms (TSCC) among adolescents sexually abused (SA) online and offline

	No SA (a) N = 4185–4223		SA only ouside the internet (b) N = 1073–1091		SA only on the internet (c) N = 15		SA both outside and on the internet (d) N = 17		Stat sign
	M	SD	M	SD	M	SD	M	SD	One way ANOVA with Bonferroni correction
TSCC									
Anxiety	4.20	3.63	6.82	4.53	7.20	6.38	9.41	5.35	a/b .000, a/c .015, a/d .000, b/c ns, b/d .035, c/d ns
Depression	4.61	4.14	7.59	5.14	8.33	6.54	13.29	6.65	a/b.000, a/c .006, a/d .000, b/c ns, b/d .000, c/d .008
Anger	3.75	3.86	5.74	4.57	8.40	7.00	7.59	4.89	a/b .000, a/c .000, a/d .001, b/c ns, b/d ns, c/d ns
Posttraumatic stress	5.50	4.57	9.26	5.82	9.87	6.60	13.47	7.43	a/b .000, a/c .003, a/d .000, b/c ns, b/d .002, c/d ns
Dissociation	5.48	4.55	8.29	5.45	8.53	6.83	12.88	6.33	a/b .000, a/c ns, a/d .000, b/c ns, b/d .000, c/d ns
Sexual	2.02	2.35	3.10	2.77	4.80	4.90	4.65	3.10	a/b .000, a/c .000, a/d .000, b/c .045 b/d ns, c/d ns
Critical items	1.37	2.20	3.12	3.12	4.53	5.91	6.18	4.16	a/b .000, a/c .000, a/d .000, b/c ns, b/d .000, c/d ns
Total	26.80	18.91	41.44	23.13	49.07	37.62	62.18	27.78	a/b .000, a/c .000, a/d .000, b/c ns, b/d .000, c/d ns

### Risk behaviors, internet use and pornography consumption

Table 5 shows that the index group students reported significantly different online behaviors than those in the reference group. The difference was not significant with respect to time spent online but was significant with respect to what was being engaged in online. All of the following behaviors were more common in the index group than in the reference group: had more often during the preceding year shared contact information (43.8% vs. 12.0%, *p* < .001), looked for someone to talk sex with (38.7% vs. 3.8% %, *p* < .001) or had sex with (35.5% vs. 3.5%, *p *< .001), sent nude pictures (71.9% vs. 24.4%, *p* < .001) and posted nude pictures on a community or internet site (25% vs. 1.9%, *p* < .001). They also had been offended far more often by crude sexual language online (28.1% vs. 3.8%, *p* < .001).

**Table 5:** Online sexual abuse—risk behaviors, internet behavior and pornography consumption

	Not sexually abused on the internet n = 5498–5663		Sexually abused on the internet n = 31–32		p-value^a^
	n	%	n	%	*p*
Alcohol use last year					
Drink 2–3 times or more per month	1944	34.2	11	34.4	ns
Drug use ever					
Ever used drugs, including cannabis	1316	23.3	15	48.4	.001
Sexual debut (mean age/SD)	15.55/2.50		15.78/1.78		ns^b^
Number of sexual partners					
≥ 6 partners	950	25.5	9	37.5	ns
Time spent per day					
Computer/tablet ≥ 5 h	1275	22.2	9	28.1	ns
Social media ≥ 5 h	839	14.8	9	28.1	.035
Mobile phone ≥ 5 h	1813	32.0	13	40.6	ns
Internet behavior last year					
Shared your e-mail, telephone number or address to someone you only knew through the internet					
Yes, several times	682	12.0	14	43.8	< .001
Looked for someone online to talk, sex with					
Yes, several times	215	3.8	12	38.7	< .001
Looked for someone online to have sex with					
Yes, several times	200	3.5	11	35.5	< .001
Been offended by crude sexual language when you chatted with a person you only knew through the internet					
Yes, several times	212	3.8	9	28.1	< .001
Sent nude pictures	1385	24.4	23	71.9	< .001
Posted nude pictures (community/internet site)	109	1.9	8	25.0	< .001
Pornography					
Have you ever looked at pornography					
Yes	3865	68.1	23	71.9	ns
Have often have you looked at pornography the last 12 months					ns
Not at all	34	0.9	0	0.0	
1–2 times	1131	29.3	3	13.0	
Sometimes each month	898	23.2	9	39.1	
Sometimes each week	1196	30.9	9	39.1	
More or less daily	606	15.7	2	8.7	

^a^ p-value based on Chi square or Fisher’s exact test

^b^ p-value based on Mann–Whitney’s U-test

The experience of having ever used drugs was more common in the index group (48.4% vs. 23.3%, *p* < .001) but alcohol consumption did not differ between the index group and the reference group. There were no significant differences between the groups in relation to age of sexual debut, number of sexual partners, or extent of consumption of pornography.

### Multiple logistic regression analyses

Stepwise multiple logistic regression analyses for Tables 1, 2, 3 and 5, 6 separately revealed 11 variables that could be analyzed to produce a final model with five variables, Table 6. In the final model experiences of abuse such as penetrative sexual abuse (OR 3.68, CI 1.58–8.58) and threats of being hit (OR 2.33, CI 1.04–5.24) were significantly associated with being sexually abused online. Risky internet behavior such as looking for someone online to talk sex with (OR 6.52, CI 2.73–15.57) and posting nude pictures on a community or internet site (OR 4.74, CI 1.70–13.16) were also highly associated with having been sexually abused online. Finally, the subscale depression was also significantly associated with being sexually abused online (OR 1.11, CI 1.04–1.17).

**Table 6:** Online sexual abuse—forward StepWise logistic regression to identify important variables among each block of variables

Block	Variables identified as statistically significant within each block	OR (95% CI)
Table 2a	Family financial situation	
	Poor	1.78 (.78–4.02)
	Don’t know	3.53 (1.04–11.95)
Table 2b	Any sexual abuse	2.64 (1.02–6.18)
	Penetrative sexual abuse	2.76 (1.02–7.50)
	Threats of hitting	3.60 (1.68–7.23)
Table 3	PBI overprotection, father	1.07 (1.02–1.12)
	TSCC depression	1.15 (1.06–1.26)
	TSCC sexual anxiety	1.35 (1.14–1.61)
Table 5	Looked for someone online to talk sex with	4.80 (1.66–13.88)
	Been offended by crude sexual language when you chatted with a person you only knew through the internet	5.12 (1.78–14.67)
	Posted nude pictures (community/internet site)	5.05 (1.60–15.87)
Final model	Penetrative sexual abuse	3.68 (1.58–8.58)
	Threats of hitting	2.33 (1.04–5.24)
	TSCC depression	1.11 (1.04–1.17)
	Looked for someone online to talk sex with	6.52 (2.73–15.57)
	Posted nude pictures (community)	4.74 (1.70–13.16)

Variables to be included in a “final model” was reduced by performing stepwise multiple logistic regression analyses for each table separately

## Discussion

To our knowledge, this study is the first to study adolescents with experiences of online sexual abuse by a person they had met online and where they had felt persuaded, pressed or coerced. The results of the study can be summarized in four main findings.

*First*, the study showed that most sexual contacts online were positive experiences with persons of about the same age or only slightly older. However, previous studies have shown that having a sexual relationship with a person met online can be viewed as a risk behavior since this kind of contact increases the risk of facing negative consequences later, for example receiving unwanted sexual approaches [12]. Similar reasoning has been put forward by Baumgartner et al. [14, 26] in defining online sexual risk behaviors as the exchange of intimate sexually insinuating information and material with someone only known online. In the current study, 5.8% of the adolescents had had sexual experiences online with a person they had only met online, and of those, 9.7% reported that they had been persuaded, pressed or coerced meaning that they, by definition, had been sexually abused online. Girls were more often the victims and for girls, the perpetrators were generally older.

*Second*, there were no significant differences in socio-demographic background between the index group and the reference group. This result can be compared to studies on children victims of online grooming [13] or adolescents sending nude images [5] were it was also found that the socio-demographic background did not differ from children without these experiences.

*Third*, the adolescent victims of online sexual abuse had backgrounds with significantly more numerous and/or varied experiences of different forms of abuse including physical, psychological as well as sexual abuse, especially penetrative sexual abuse than those who had not been victims of online sexual abuse. Earlier findings indicate that the more severe the form of sexual abuse the more serious the subsequent associated health issues will be, with penetrating child sexual abuse at the upper end of the scale of severity [27]. This study underlines these earlier findings but also adds to our knowledge that online abuse per se is also associated with poor health, low self esteem and a poorer relationship between parent and child. As concerns health, as measured by TSCC, online sexual abuse only was associated with poorer health, at least on the same level as offline sexual abuse only, with those students who had been sexually abused both online and offline scoring highest, supporting the polyvictimization model [28].

These results are also supported by earlier studies [15, 16, 29–31] stating that online sexual victimization, also including cyberbullying, are associated with adverse emotional and psychological consequences. In the current study, the final multiple logistic regression model showed that online sexual abuse was strongly associated with depression. This is in line with the results from studies focusing on youth who had sent sexual pictures (sexted), where both Van Ouystel et al. [32] and Dake et al. [33] found an association between sexting and depression. In the study by Temple et al. [34] associations were also found between sexting and depression in their unadjusted models, but not when prior sexual behavior, age, gender, race, ethnicity, and parental education had been adjusted for. It is, however, important to bear in mind that the studies referred to above do not examine if the motivation factor for sending the images was, for example, sending the image just for fun and with no negative consequences afterwards or if it was because of coercion leading to the taking and sending of the image.

*Fourth*, adolescents abused online also had more online risk behaviors such as sharing personal information significantly more often, looking for someone online to talk sex with, or posting nude pictures on a community site. These behaviors might increase the risks of later being a victim of online sexual abuse [17].

The results in the study should be read in light of the following limitations. The response rate was rather low at 59.7%. Part of this can be explained by the fact that on a typical day 10% of students of this age are absent from school. An assumption is that the absent group probably would have added some individuals to the index group and thereby affected the results slightly, since people dropping out from research more often come from families with poorer support and are more often burdened with psychosocial health issues and lower motivation to participate in school surveys [35]. On the other hand, other studies that have found little evidence for substantial bias as a result of nonparticipation [36]. Recall bias is always a limitation in questionnaire-based studies, as is the question of whether the answers are trustworthy. All answers were reviewed before the analyses and 34 questionnaires were excluded due to unserious answers. Another limitation is the small size of the index group which may cause low statistical power. The main concern regarding study power arises when the index group is separated into two groups. When comparing these two groups to the reference group, statistical significance is detected, even though the power is well below 80%. However, in all but one comparison between the two subgroups (SA internet, SA offline and internet) no statistical difference was detected. Having a larger power would probably result in more statistically significant findings. The implication of the low power is that we underestimate rather than overestimate the presence of actual differences between the groups.

Finally, the index question did not contribute to any additional probing to determine what online sexual activities or sexual abusive behaviors respondents might be referring to when they endorsed these items, nor did it allow them to describe the behavior further. It would have been conceptually interesting to have a fuller description and examples from respondents.

## Conclusions

The socio-demographic background of the adolescent victims of online sexual abuse in the current study did not differ from the background of adolescents without this experience, but significant differences were found in relation to their prior experience of different forms of abuse indicating that they belong to a polyvictimized group. Together with risky online behavior, the poorer psychological health in combination with poor relationships with parents and low self-esteem might increase the vulnerability of these individuals to having sexual contact online and having that contact with people unknown to them who might then abuse them. It is also plausible to think that poorer health can be a consequence of the abusive online experiences but also the other way around since we can’t establish the causality in this kind of cross-sectional study. The study demonstrates the importance of viewing online sexual abuse as a serious form of sexual abuse even if the victim and perpetrator have not met outside the internet. Professionals meeting these children need not only to focus on their psychological health as indicated by symptoms of trauma and depression but also must screen for online behavior, online abuse and other forms of previous abuse.

## Data Availability

Not applicable.
